# A Foundational Shift in Models for Enzyme Function

**DOI:** 10.1021/jacs.5c02388

**Published:** 2025-04-25

**Authors:** Judith P. Klinman, Susan M. Miller, Nigel G. J. Richards

**Affiliations:** ^†^Department of Chemistry, ^‡^California Institute for Quantitative Biosciences, and ^§^Department of Molecular and Cell Biology University of California, Berkeley, California 94720, United States; ∥ Department of Pharmaceutical Chemistry, University of California, San Francisco, California 94158, United States; ⊥ Foundation for Applied Molecular Evolution, Alachua, Florida 32615, United States; # School of Chemistry, Cardiff University, Cardiff CF10 3AT, United Kingdom

## Abstract

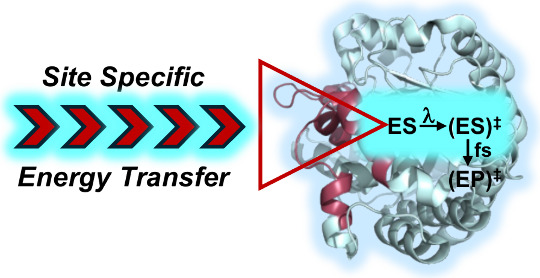

This Perspective addresses the unresolved, and still hotly contested, question of how enzymes transition from stable enzyme–substrate (ES) complexes to successful, femtosecond barrier crossings. By extending Marcus theory to enzyme-catalyzed reactions, we argue that environmental reorganization of the protein scaffold, together with associated water molecules, achieves the intersection of reactant and product potential energy surfaces. After discussing the experimentally demonstrated importance of reduced activation enthalpy in enzyme-catalyzed transformations, we describe new methodologies that measure the temperature dependence of (i) time-averaged hydrogen/deuterium exchange into backbone amides and (ii) time-dependent Stokes shifts to longer emission wavelengths in appended chromophores at the protein/water interface. These methods not only identify specific pathways for the transfer of thermal energy from solvent to the reacting bonds of bound substrates but also suggest that collective thermally activated protein restructuring must occur very rapidly (on the ns–ps time scale) over long distances. Based on these findings, we introduce a comprehensive model for how barrier crossing takes place from the ES complex. This exploits the structural preorganization inherent in protein folding and subsequent conformational sampling, which optimally positions essential catalytic components within ES ground states and correctly places reactive bonds in the substrate(s) relative to embedded energy transfer networks connecting the protein surface to the active site. The existence of these anisotropic energy distribution pathways introduces a new dimension into the ongoing quest for improved *de novo* enzyme design.

## 1 Introduction

- Expanding our understanding of the underlying physical properties of enzyme function provides new design principles.
- Findings from experimental probes are essential to guide future computational predictions.

After almost 80 years, Pauling’s proposal of “enhanced binding between enzymes and their activated substrate”^1,2^ remains the dominant model for the origin of enzyme catalysis.^3−5^ Yet, after so many decades of investigation, a nagging question remains: Why is the successful *de novo* design of enzymes limited to only a small number of relatively simple chemical reactions? An earlier Perspective from this laboratory stressed the need for integrating experimental findings with computational studies to address this question.^6^ During the intervening years, the sophistication and use of computation in understanding enzymes has only increased,^7^ but, even now, “designer enzymes” remain unable to reproduce the extraordinarily complex transformations and rate accelerations achieved by Nature.^8^ This leaves open the question of whether we understand the physical principles underpinning enzyme function at a level that permits any successful application of computation toward obtaining enzymes by *de novo* design.^9^ If we do indeed lack this understanding, then new experimental approaches will be a cornerstone in driving the conceptual developments needed to advance the field.^10,11^

That protein dynamics is important in influencing enzyme regulation and function is increasingly accepted,^12−14^ despite a lack of consensus about what the term dynamics means in enzyme catalysis.^15,16^ In this Perspective, we limit our discussion to motions within the protein scaffold that impact access to activated complexes. As essentially all enzymes (with rare exceptions)^17,18^ are thermally activated, we also seek to move beyond the long-standing problem of how enzymes lower the activation free energy,^19−21^ Δ*G*^⧧^, to focus on reductions in Δ*H*^⧧^ while posing the question: *Can we develop experimental probes that provide a molecular description of the events that convert a ground state enzyme–substrate complex to its activated complex?* As will be shown, comparing the time and temperature dependencies of enzyme chemistry to time and temperature-dependent motions in protein scaffolds has uncovered long-range dynamical networks that play essential roles in facilitating chemical transformations at the active site.

## 2 Environmental Reorganization in Condensed Phase and Enzymes

- Environmental reorganization controls the barrier to chemical reactivity in condensed phase reactions.
- For enzyme reactions, barrier crossings are linked to motions within the protein scaffold.

### 2.1 Condensed Phase Reactivity

In introductory chemistry, reactivity is often taught in the context of gas phase collisional encounters, with activated complexes arising from conversion of kinetic energy into energized vibrational states.^22^ This model gives a molecular explanation for the Arrhenius equation,^23^ which is an empirical representation of the activation energy of a reaction with a pre-exponential frequency factor generally assigned an upper limit of 10^13^ s^–1^. Eyring’s formulation provides a clear-cut separation of activation free energy into enthalpic and entropic contributions (eq 1):^24^
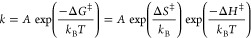
1where Δ*H*^⧧^ = *E*_a_ – *RT* and the pre-exponential factor *A* = *k*_B_*T*/*h*.^25^ The description of unimolecular reaction rates in condensed phase is different and requires special consideration. First, reactants in solution must “find each other” via diffusional encounters within a solvating medium. Subsequent to the formation of an “encounter complex”, changes in solvent organization are needed to stabilize the altered charge distributions in the activated complex.^26^ The chemical reaction takes place within a large number of equilibrating reactant and solvent conformational states. As there is no time for conformational re-equilibration during the femtosecond time scale of transition state crossings, multiple coordinates connect corresponding vibrational levels in the reactant(s) and activated complex. This model leads to an alternate formulation of the rate constant (eq 2):
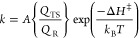
2where *Q*_TS_ and *Q*_R_ are partition functions (translational, rotational, vibrational) for the activated complex and reactant(s).^27^ When energy spacings in the activated complex and reactant(s) are similar, the entropic term is generally small, focusing attention on Δ*H*^⧧^ in the rate constant expression.

### 2.2 Conceptual Framework for Reaction Barriers in Solution

**Figure 1:**
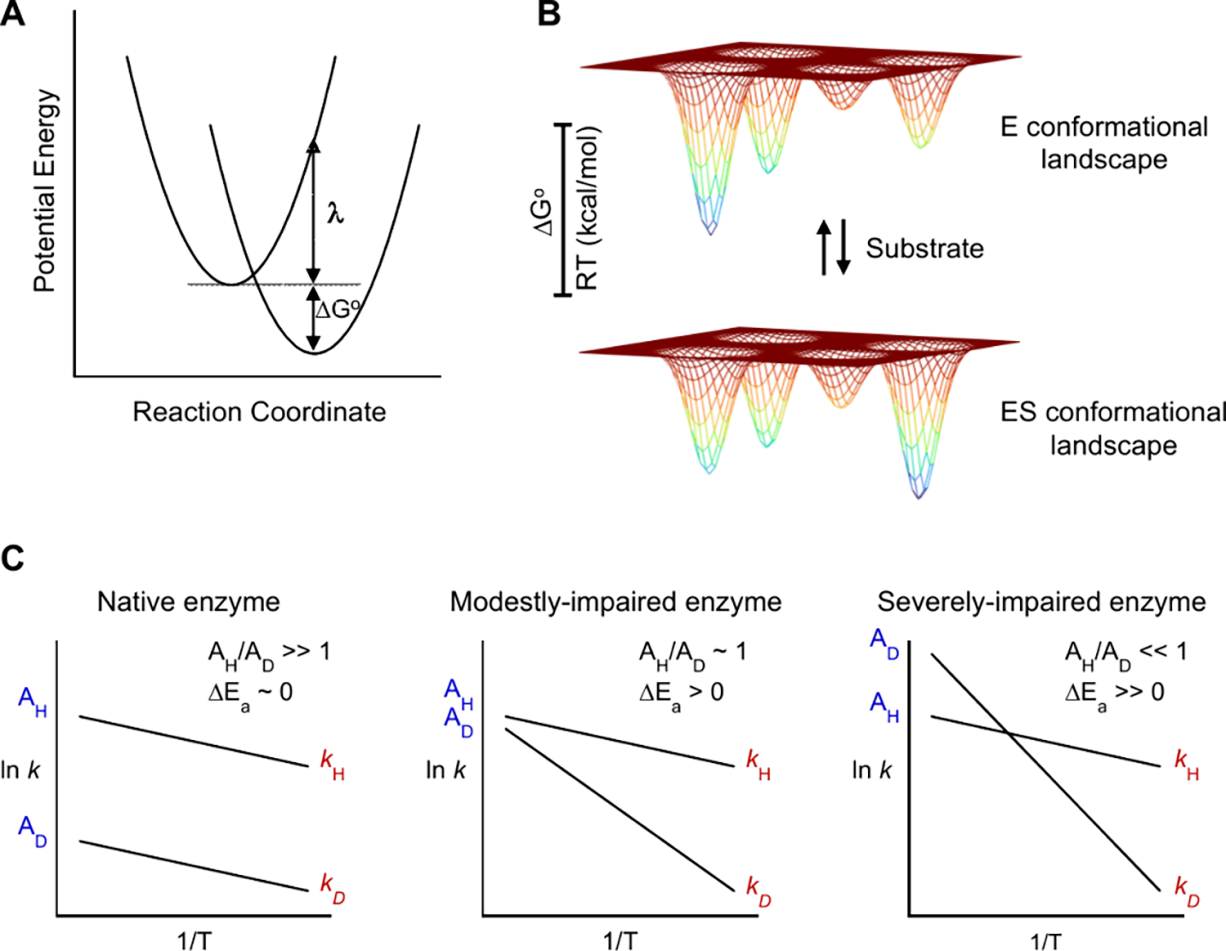
(A) Importance of environmental reorganization as a central feature in condensed phase reactivity, as defined by λ.^28−30^ (B) Proteins undergo rapid sampling of a wide range of ground state structures near room temperature. The distribution among these protein substates is sensitive to substrate or other ligand binding through a process of conformational selection.^33^ (C) Temperature dependence of the primary kinetic isotope effect for enzyme-catalyzed C–H cleavage varies with enzyme impairment and can provide strong support for models of H-transfer by tunneling.^34^ See Section 3.

The central role of solvent in barrier crossing is captured by Marcus theory,^28−30^ which relates activation free energy, Δ*G*^⧧^, to thermodynamic driving force, Δ*G*°, and λ, the energetic barrier associated with environmental reorganization (eq 3):
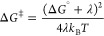
3Here λ is defined as the energy difference between reactant ground state and the point on the reactant potential energy surface (PES) that corresponds to the stable configuration/solvation of product (Figure 1A). Marcus theory does not normally consider ground state vibrational levels and is formally valid only when the vibrational energy spacings in the activated complex and reactant(s) are similar (Δ*S*^⧧^ is small). Movement along the reaction coordinate requires reorganization of the surrounding solvent molecules to complement changes in the reactant at the activated complex and this becomes a dominant contributor to the magnitude of λ.^31^ These models assume that reactants first diffuse together to form a precursor complex, representing a preorganization that is differentiated from the reorganization energy (λ in eq 3).^32^

The Marcus formalism does not address the problem of *how* the reactants gain the energy to pass through the activated complex. For most reactions, there is a substantial change in charge distribution as reaction proceeds along the reaction coordinate, leading to a strong coupling of the altered reactant electrostatics with the dipoles of surrounding solvent molecules, particularly in the case of water.^26^ For example, Hynes and co-workers used molecular dynamics (MD) simulations to map changes in energy along the reaction coordinate of the S_N_2 reaction between chloride anion and methyl chloride in water.^35^ Their calculations indicated that changes in the energy of water, associated with solvent reorganization, were the primary source of energy needed by the reactants to attain the activated complex. Further, the time scale of water reorganization was longer than that needed for barrier crossing, making it the primary origin of the reaction rate.

### 2.3 Differentiating Enzyme and Small Molecule Reactivity: Importance of Decreasing Δ*H*^⧧^ for Enzyme Catalysis

Enzyme-catalyzed reactions differ from those in solution by occurring within a preorganized cage of the protein scaffold. Traditionally, a large protein scaffold was ascribed the role of *insulating* bound substrate(s) from the solvent bath.^36−38^ Moreover, enzymes were generally assumed to be static,^39^ with the exception of the conformational changes necessary for substrate ingress, product egress, allostery and loop closures.^40,41^ It is now clear that intrinsic enzyme fluctuations play an important role in function, particularly through distributed conformational ensembles, arising from small variations in atomic position,^42,43^ with energies readily accessible near room temperature (RT, 0.6 kcal/mol at 298 K) (Figure 1B). Such sampling locates suitably positioned and solvated enzyme–substrate (ES) ground states for progression to the activated complex.^44^ Note again that this sampling is analogous to the preorganization factor that accompanies Marcus theory and is distinguished from the environmental reorganization (λ in eq 3) that *drives* the conversion of optimized ground states to their activated complexes.

The parameters *T*Δ*S*^⧧^ and Δ*H*^⧧^ are known for many enzyme-catalyzed reactions (Table 1). When changes in heat capacity are negligible over the temperature range, a plot of ln(*k*/*T*) vs 1/*T* is linear, with a slope of −Δ*H*^⧧^/*k*_B_ (eq 1).^45^ With a number of interesting exceptions,^46^ this assumption holds, and ln(*k*/*T*) is well approximated by ln(*k*) for the temperature range where enzymes function. Interpreting experimental Δ*H*^⧧^ values for enzyme reactions can be complicated by factors having varying degrees of potential impact including changes in heat capacity of the system, protein unfolding at elevated temperature, temperature-dependent population changes in catalytically inactive conformations of the ES complex,^47^ altered p*K*_a_ values for ionizable groups in the buffer, protein and substrate(s), and changes in rate-determining steps.^48^ The importance of finding a temperature regime that produces linear Arrhenius plots with close to a constant rate-limiting step cannot be overemphasized. Data need to be collected with extreme care, accompanied by many controls, to arrive at interpretable activation parameters.

**Table 1:** Experimentally Measured Δ*H*^⧧^ and Δ*S*^⧧^ Contributions in Enzyme-Catalyzed Reactions

Enzyme	*k*_cat25_ (s^–1^)	Δ*H*_cat_^⧧^^a^^,^^b^ (kcal/mol)	*T*Δ*S*_cat25_^⧧^ (kcal/mol)	Cat. refs	*k*_non25_ (s^–1^)	Δ*H*^⧧^_non_^a^(kcal/mol)	Noncat. refs
yeast OMP decarboxylase	15	11.2	–4.7	(49)	2.8 × 10^–16^	37.1	(50)
staphylococcal nuclease	95	10.8	–3.9	(51)	1.7 × 10^–13^	29.5	(51)
urease	34,900	9.9	–1.4	(52)	1.2 × 10^–11^	22.9	(53)
adenosine deaminase	160	10.9	–3.6	(54)	1.8 × 10^–10^	22.0	(55)
cytidine deaminase	299	14.9	0.9	(56)	2.7 × 10^–10^	22.1	(56)
chorismate mutase	50	12.7	–2.5	(50, 57)	2.6 × 10^–5^	20.7	(58)
fumarase	3,162 (F→M)	8.2	–4.5	(59)	5.2 × 10^–14^	28.9	(60)
	1,778 (M→F)	13.9	0.8				
mandelate racemase	889	15.4	2.0	(61)	3.0 × 10^–13^	31.9	(62)
carbonic anhydrase C	1 × 10^6^	7.5	–1.8	(63)	3.6 × 10^–2^	17.3	(63)
dihydroorotase	0.40	12.3	–5.0	(64)	3.2 × 10^–11^	24.7	(64)
chloroacrylate dehalogenase	3.8	9.4	–7.2	(65)	2.2 × 10^–12^	26.7	(65)

a Approximate errors in Δ*H*^⧧^ of ∼2 kcal/mol.

b The fractional contribution of Δ*H*^⧧^ to Δ*G*^⧧^, Ave = 0.77 ± 0.18 (5 SD, 99.9%).

Seminal experiments by Wolfenden for enzyme-catalyzed reactions and their uncatalyzed counterparts show that enzymes decrease Δ*H*^⧧^ significantly relative to the cognate solution-phase reaction with relatively small impacts on *T*Δ*S*^⧧^ (Table 1).^19,49−65^ The average experimental Δ*H*^⧧^ for enzymatic transformations is ∼10 kcal/mol, quantitatively distinguishing this barrier from distributed RT conformational sampling. Computer simulations, principally by Warshel,^66^ have independently made a convincing case for the importance of electrostatic interactions in lowering Δ*H*^⧧^ for enzyme-catalyzed reactions. The importance of Δ*H*^⧧^ in enzymatic catalysis was also noted early by Bruice and Benkovic,^67,68^ leading them to propose enthalpically driven, near attack conformations that poise the reactant(s) for access to the activated complex.

## 3 Anomalous Kinetic Isotope Effects Implicate Protein Scaffold Motions in Generating the Activated Complex

- Temperature-independent kinetic hydrogen isotope effects indicate transient active site compaction in enzymes catalyzing C–H activation.
- The key role of protein scaffold motions in reducing donor–acceptor distances is captured by vibronically non-adiabatic H-tunneling models.

### 3.1 Anomalous Kinetic Isotope Effects (KIEs)

KIE measurements can distinguish chemical steps from protein conformational changes and/or substrate binding/product release steps,^69^ and play an important role in analyzing enzymatic behavior and mechanism.^70,71^ In “semi-classical” descriptions of KIEs, the PES is independent of isotopic substitution. Differences in reaction rate for isotopomers arise exclusively from isotopically dependent vibrational modes in the ES and activated complexes.^72^ However, primary deuterium KIEs in some enzymatic reactions have been observed to have magnitudes that greatly exceed semiclassical limits,^73^ to be temperature-independent at, or near, room temperature (Figure 1C),^74,75^ and to show comparative multiple secondary isotope effects for (H,D) and (D,T) transfer that differ from predicted reduced mass relationships.^76^ Bell’s model rationalized some of this behavior by incorporating a tunneling correction factor into transition state theory-based rate equations.^77^ Variational transition state theory was subsequently developed by Truhlar and co-workers,^78^ in which the traditional reaction barrier is maintained while introducing isotope-dependent crossing points (H-transfer below that for D-transfer). The need for yet another approach, however, was compellingly demonstrated by *the observation of parallel lines in Arrhenius plots for H- and D-transfer, indicating independent reaction coordinates for the isotopic and temperature dependencies of reaction* (Figure 1C, left frame).^34,74^

### 3.2 Vibronically Non-adiabatic Models for Hydrogen Transfer

Analytical equations able to accommodate the deviant behaviors summarized above were independently put forth by Borgis and Hynes,^79^ and by Kuznetsov and Ulstrup,^80^ with further refinements introduced later by Hammes-Schiffer,^81−83^ and this laboratory.^84−87^ These treatments extend the Marcus model for electron transfer by describing the movement of hydrogen as a wave. Here we discuss one such representative analytical expression (eq 4),^88^ derived for non-adiabatic hydrogen atom tunneling where the electron and proton move to different acceptors; the impact of proton donor–acceptor distance (DAD) is included in the integral component of the rate constant:
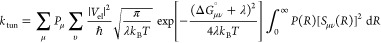
4Here *P*_μ_ is the Boltzmann probability of reaction occurring from vibrational mode μ of reactant to a vibrational mode ν of product, and Δ*G*_μν_^°^ is the free energy difference between reactant and product states, including the vibrational modes of reactant (μ) and product (ν), which varies with the reaction of interest. For hydrogen transfer catalyzed by soybean lipoxygenase (see below), protium wave function overlap is dominated by the (0,0) modes, although deuterium tunneling depends, to some extent, on vibrationally excited states.^84^ The exponential term containing λ dominates the temperature dependence of the rate constant.

The continuous integral in eq 4 shows how hydrogenic wave function overlap, *S*_μν_, becomes less probable as *R* increases. In the simplest case,^88^ protein motions of mass (*M*) and frequency (Ω) that generate different *R* values are modeled as a classical harmonic oscillator (eq 5):
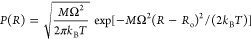
5The importance of Ω in moderating *P*(*R*) is clearly evident, where higher frequencies reflect more compact active sites and reduced distance sampling. In native enzymes capable of generating a highly reorganized ground state,  ≫ 1, meaning the exponential term has a small impact on *k*_tun_. This finding explains the parallel lines in Arrhenius plots for protium- and deuterium-transfer reactions catalyzed by evolved native enzymes,^34,89,90^ (Figure 1C, left) where temperature- and isotope-dependent terms reside in different coordinates. For less optimized enzymes, or impaired variants that are generally obtained by site-specific mutagenesis, DAD sampling increases, due to a decrease in Ω that accompanies the loss of precise binding in ground state complexes (Figure 1C, middle and right).^84,91^ The impact of decreasing Ω is greater for deuterium than protium because of its increased mass and shorter wavelength, as represented by the negative sign for (α_H_^2^ – α_D_^2^) in eq 6:^92,93^
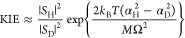
6The intrinsic source of the KIE is the square of the S_H_/S_D_ ratio, arising from the difference in wave function overlap for protium and deuterium at *R*_o_. When an enzyme becomes optimized and reactant(s) are confined within the active site (Ω large), a temperature-independent KIE can result. This behavior is beautifully illustrated by experimental studies of hydride tunneling in dihydrofolate reductase (DHFR), where a “primitive” DHFR variant exhibited temperature-dependent KIEs that became temperature-independent following the use of directed evolution methods to give a variant in which the active site and *k*_cat_ were optimized.^94^ The correlation between the compactness of the active site and catalytic efficiency is also seen in similar studies of designer enzymes, such as a retroaldolase^95^ and a Kemp eliminase.^96,97^

Overall, the magnitude and temperature dependence of experimental KIEs are well reproduced by eqs 4–6, indicating the robustness of the non-adiabatic approximation. For enzyme variants with altered temperature dependencies of their KIEs, i.e. *E*_a_(D) > *E*_a_(H) (Figure 1C), we note it is conceptually possible to substitute the DAD sampling mode in eq 5 by an isotope-dependent environmental reorganization energy term, λ′. Two significant findings emerge from fitting experimental data to eqs 4–6. First, scaffold protein motions are shown to play a role in tuning DADs to enhance barrier crossing.^84,98^ Second, numerical estimates based solely on *k*_tun_ (eq 4) greatly exceed measured values, *k*_obs_.^99^ The latter discrepancy reflects the fact that tunneling is a *very rare event*, *dependent on transiently achieved high energy environmental configurations* (eq 7):
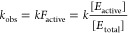
7where *F*_active_ ≪ 1. We will return to this important property below.

## 4 Experiment Discovers Catalytically Relevant Thermal Networks

- Temperature-dependent hydrogen–deuterium exchange measurements identify energy conduits that reach from protein/solvent interfaces to the active site.
- Thermal networks are unique to the chemical reaction being catalyzed and are not conserved within a protein family.

### 4.1 Spatial Resolution of Functionally Relevant Protein Regions by Temperature-Dependent Hydrogen–Deuterium Exchange Mass Spectrometry (TDHDX-MS)

**Figure 2:**
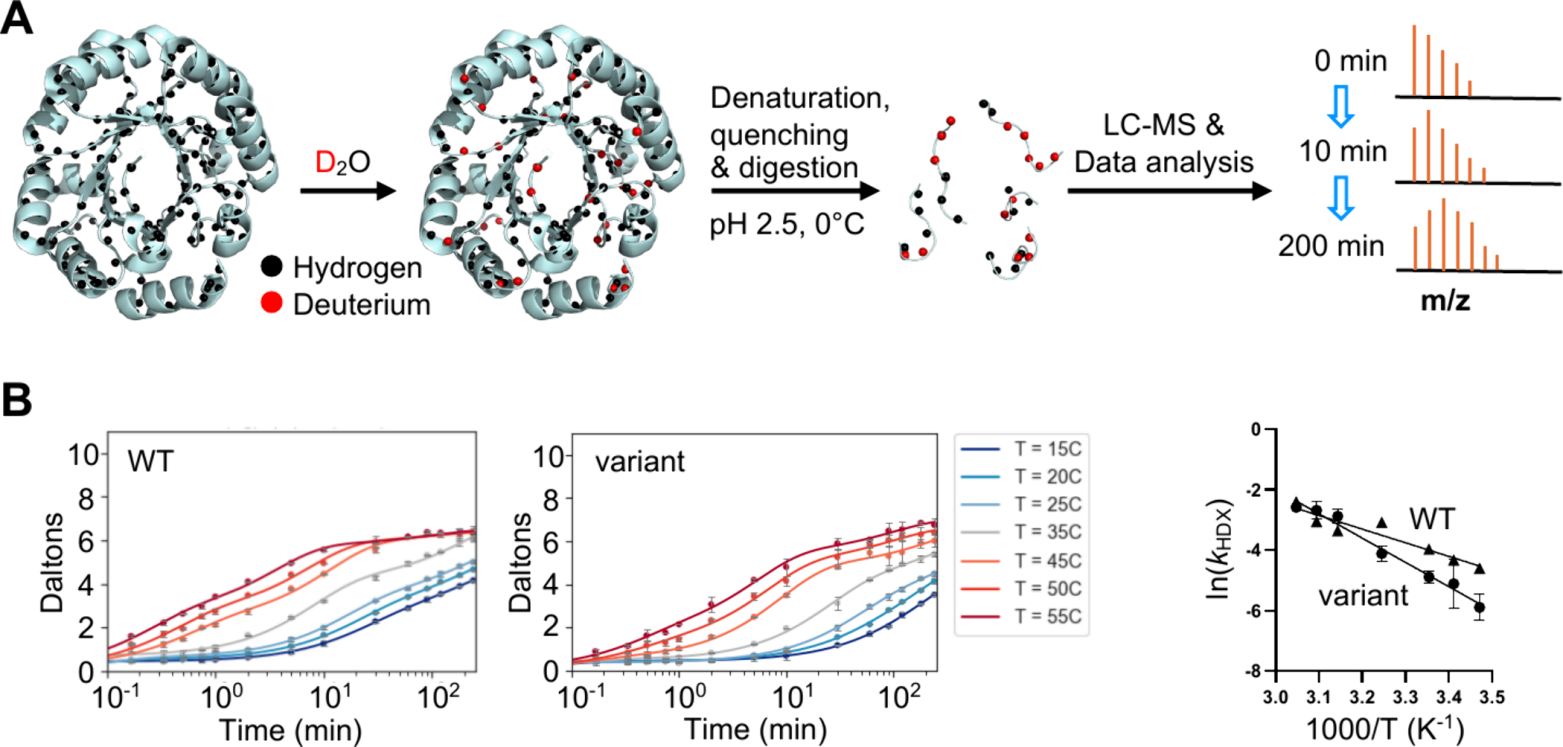
Extending HDX-MS methodology to multiple temperatures identifies changes in local protein flexibility, *E*__a__(*k*_HDX_) following insertion of site-specific mutations that alter the activation energy for catalysis, *E*_a_(*k*_cat_). (A) Canonical protocol for conducting HDX-MS involves time-dependent monitoring of deuterium uptake into backbone amides of a folded protein vs time. Mass spectrometric monitoring is performed on isolated peptides obtained under low temperature and pH conditions to minimize back exchange of incorporated deuterium. (B, left) Plots of time and temperature changes for deuterium uptake, illustrated for WT OMPDC and a single site variant with altered *E*_a_(*k*_cat_). (B, right) Arrhenius plots of rate constants (units of s^–1^) obtained at the indicated temperatures lead to different slopes representing the induced local change in *E*_a_(*k*_HDX_) for the function altering variant.^106^

Understanding temperature-dependent environmental reorganization within a protein scaffold provides a frame of reference for understanding how temperature impacts enzymatic activity. As a rule, globular proteins possess irregular, rugged surfaces, and the anisotropic nature of vibrational excitation pathways has been demonstrated experimentally and by computation.^100−102^ Any *a priori* pursuit of the location of functionally relevant, anisotropic motions, however, resembles the proverbial “search for a needle in a haystack”. We decided to use HDX-MS,^103−105^ which is broadly applicable to proteins of all sizes, requires relatively small amounts of material, and is capable of detecting structural and dynamical features unseen using other methodologies. In this laboratory, HDX-MS protocols start with incubating the enzyme of interest in D_2_O under canonical conditions, with measurements being collected at time intervals ranging from 10 s to 4 h (Figure 2A).

After quenching HDX by reducing temperature and pH, proteolysis converts the protein into small peptides that provide close to 100% coverage of the sequence. Mass spectrometric analysis of the mixture reveals the amount of isotopic uptake at each time point (Daltons(*t*)) for each proteolytic peptide (eq 8):^107,108^
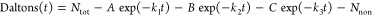
8where *N*_tot_ is the total number of exchangeable backbone amides in the specific peptide and *N*_non_ is the number of amides that do not undergo exchange with D_2_O during the designated time span. Rate constants *k*_1_, *k*_2_ and *k*_3_ are categorized as fast (>2.5 min^–1^), intermediate (0.05–2.5 min^–1^) and slow (<0.05 min^–1^). Data for *k*_1_ when H/D exchange is initiated by hand mixing (10 s) reflect very rapidly exchanging, exposed backbone amides, and are largely manifest in the *y*-axes of plots (Figure 2B, left). In our experiments, we collect data under EX-2 conditions (confirmed through time-dependent mass envelopes obtained by HDX-MS), with the primary goal of determining kinetic constants *k*_2_ and *k*_3_, either individually or as a weighted average, *k*_HDX_ (eq 9):
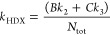
9Values of *k*_2_, *k*_3_ or *k*_HDX_ are determined across a continuum of temperatures (*T*) that preserve enzyme activity over the incubation period. Arrhenius plots (*k*_2_, *k*_3_ or *k*_HDX_ vs 1/*T*) are then constructed for individual peptides to give activation enthalpies [Δ*H*(*k*_2_), Δ*H*(*k*_3_) or Δ*H*(*k*_HDX_)] for each peptide located at a defined position within the folded protein (Figure 2B). For simplicity, we focus on *k*_HDX_ in the following analysis.

At a given temperature (*T*), the observed rate constant *k*_HDX_(*T*) is the product of the intrinsic rate constant *k*_int_(*T*) for HDX in backbone amide groups that are fully exposed to D_2_O and *K*_open_(*T*), the equilibrium constant for the local unfolding needed to expose the N–H bond to solvent (eq 10):
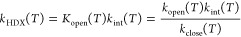
10where *k*_open_(*T*) and *k*_close_(*T*) are the rate constants determining *K*_open_(*T*). Under the EX-2 condition, *k*_close_(*T*) ≫ *k*_int_(*T*), and the enthalpic contribution to *k*_HDX_ (determined from an Arrhenius plot) can be parsed into two components (eq 11):
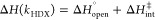
11The magnitude of Δ*H*_int_^^⧧^^ is approximately 17 kcal/mol under a variety of conditions.^109^ The more interesting parameter is Δ*H*_open_^°^ because it reports on enthalpic differences in local unfolding/flexibility of a specific peptide segment within the folded enzyme.

Peptides located in thermal networks are defined by performing comparative multitemperature measurements for one, or more, site-specific variants of the enzyme (Figure 2B). Variants are chosen by replacing hydrophobic amino acids by similar side chains of reduced volume with the goal of introducing internal packing defects without altering electrostatic or H-bonding interactions (eq 12).^84^ Ideally, the turnover time for the enzyme is limited by the chemical step.
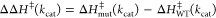
12TDHDX-MS measurements on such variants are able to probe the impact of the mutation on local protein flexibility, on the assumption that Δ*H*_int_^^⧧^^ is unchanged (with the possible exception of the altered residue and a few proximal backbone amides) (eq 13):
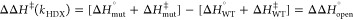
13Comparing eq 12 and eq 13 provides a linear free energy relationship, allowing the extent to which mutation impacts local flexibility, ΔΔ*H*_open_^°^ (thermodynamics), to be correlated with changes in ΔΔ*H*^^⧧^^ (*k*_cat_) (kinetics).

### 4.2 Activity-Linked, Thermal Networks Are Reaction-Dependent

**Figure 3:**
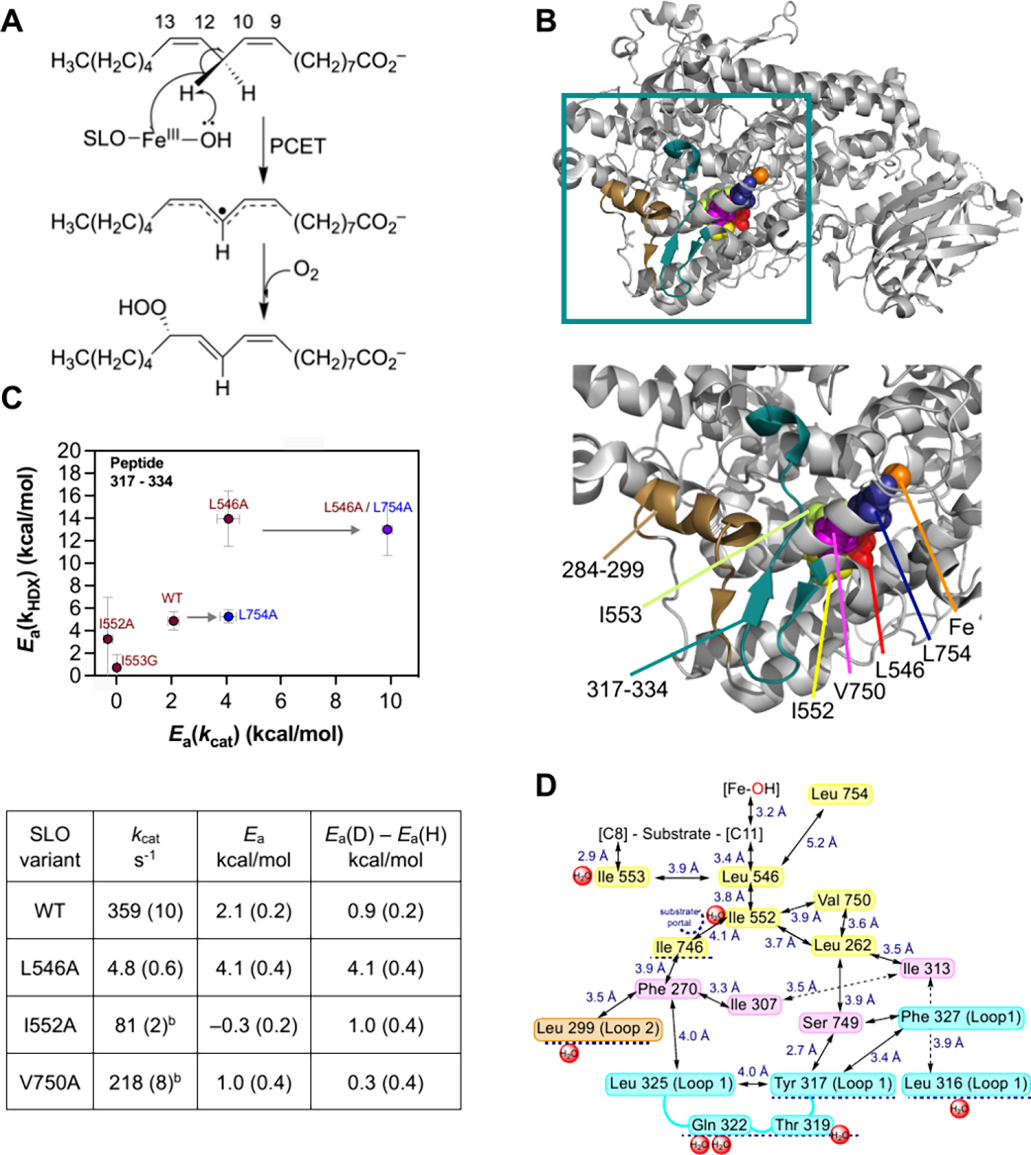
TDHDX identifies the thermal network of soybean lipoxygenase (SLO). (A) The reaction catalyzed by SLO involves a concerted proton-coupled electron transfer from C11 of substrate to the Fe(III)-OH cofactor. (B) Top shows the full structure for SLO; the enclosed box indicates the region of interest, enlarged below. The active site iron is labeled orange and the 2 peptides impacted by mutation are tan and cyan. (C) The relationship between values for *E*_a_(*k*_HDX_) and *E*_a_(*k*_cat_) for peptides 317–334 as a function of mutation site. L754 (blue) lies off the identified network.^117^ The table indicates the differential impacts of Ala on Leu546, in contact with the reactive carbon of substrate, and two remote side chains.^117^ (D) The final network indicates a radiating cone comprised of an ILV island.^107,117,118^ Yellow residues were identified through mutagenesis and/or RT X-ray studies, and those in cyan and tan are identified from TDHDX; pink presents possible intervening side chains. Panel (C) reproduced from ref (117). Copyright 2023 PNAS.

**Figure 4:**
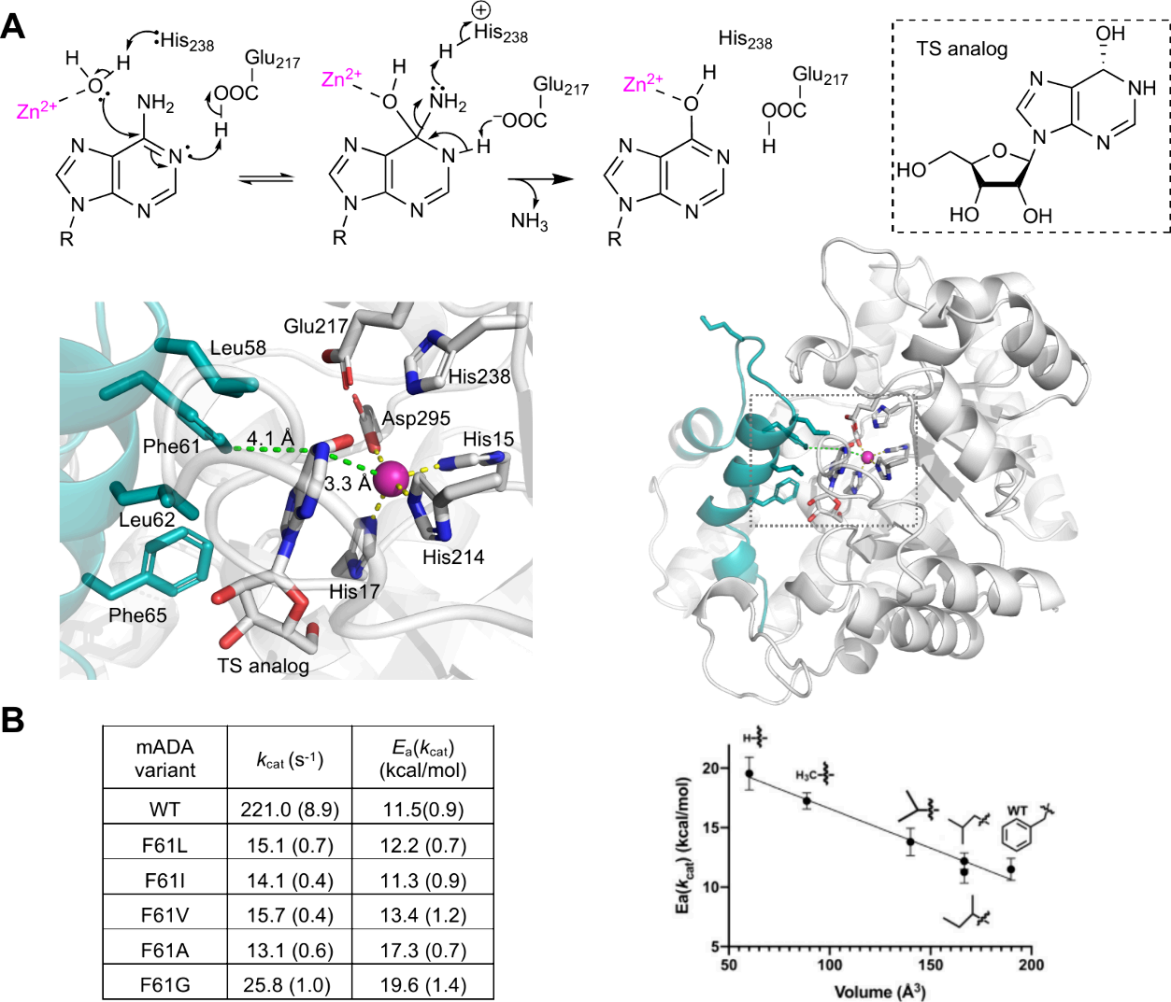
Use of TDHDX to identify the thermal network in murine adenosine deaminase (mADA). (A, top) The reaction catalyzed by mADA involves a Zn^2+^-catalyzed addition of water/hydroxide ion to the adenosine ring of substrate. (A, bottom) Structures for mADA indicating the active site (left) and its relationship to the full enzyme structure (right). (B, left) Tabulation of values for kinetically derived parameters for WT and the Phe61X series. (B, right) A plot of *E*_a_(*k*_cat_) vs the volume of the modified side chain.^54^

This laboratory has applied the TDHDX-MS protocol to a handful of enzymes, and identified, activity-linked, functionally relevant networks in all of them. For illustrative purposes, findings for two prototypic enzymes are briefly described: soybean lipoxygenase, which exhibits tunneling behavior in biological C–H activation (Figure 3A),^84^ and adenosine deaminase, a “classical system” that catalyzes the rate-limiting addition of a metal-bound hydroxide/water to a purine ring (Figure 4A).^110^ The latter is a TIM barrel enzyme (triose-phosphate isomerase), a superfamily whose members catalyze a diverse set of chemical reactions while sharing a common protein fold;^111^ indeed, close to 10% of solved X-ray and cryo-EM enzyme structures belong to the TIM barrel superfamily.^112^

#### 4.2.1 Example 1: Soybean Lipoxygenase (SLO)

The kinetic behavior of native SLO and a range of variants was the focus of an earlier Perspective highlighting primary KIEs (*k*_H_/*k*_D_ = 80–700)^34^ that greatly exceed the semiclassical limit in either enzyme-catalyzed reactions or those involving small molecules (*k*_H_/*k*_D_ = 7–10).^6^ Work in this laboratory initially applied the TDHDX protocol (Section 4.1)^107^ in a comparison of wild type (WT) SLO to two well studied variants, I553A and L546A (Figure 3).^113−116^ Changes in Δ*H*(*k*_HDX_) ∼ *E*_a_(*k*_HDX_) were restricted to 2 out of 49 peptides analyzed; both are located in a network linking the active site metal cofactor with loops at the protein/solvent interface (Figure 3B). Subsequent investigations altered residues Ile552 and Leu754 (Figure 3C); the latter resides close to bound substrate (Figure 3B). Studies of the I552A variant placed it within the energy transfer network, while the L754A variant, for which *E*_a_(*k*_HDX_) is unchanged and *E*_a_(*k*_cat_) is elevated, assigned Leu754 a role outside of the thermal network.^117^ RT X-ray studies of additional SLO variants (I552A and V750A) introduced altered side chain configurations within the assigned network (Figure 3D).^117^ This activity-linked, thermal network is built almost exclusively from aliphatic, hydrophobic side chains, located within van der Waals distance of one another and radiating in a cone-like manner from Leu546 toward the solvent.^117^

Interestingly, WT SLO exhibits a smaller *E*_a_(*k*_cat_) than most other enzymes (cf. Table 1), a finding that may reflect the challenges plants encounter in adapting to both low and high temperatures.^119^ By comparison, bacterial and human lipoxygenases show larger *E*_a_(*k*_cat_) values of 12 kcal/mol and 7–8 kcal/mol, respectively.^120,121^ Notably, TDHDX studies of the human 15-lipoxygenase uncovered an altered activity-linked, thermal network that radiates in a different direction from that seen in SLO.^121^

#### 4.2.2 Example 2: Murine Adenosine Deaminase (mADA)

**Figure 5:**
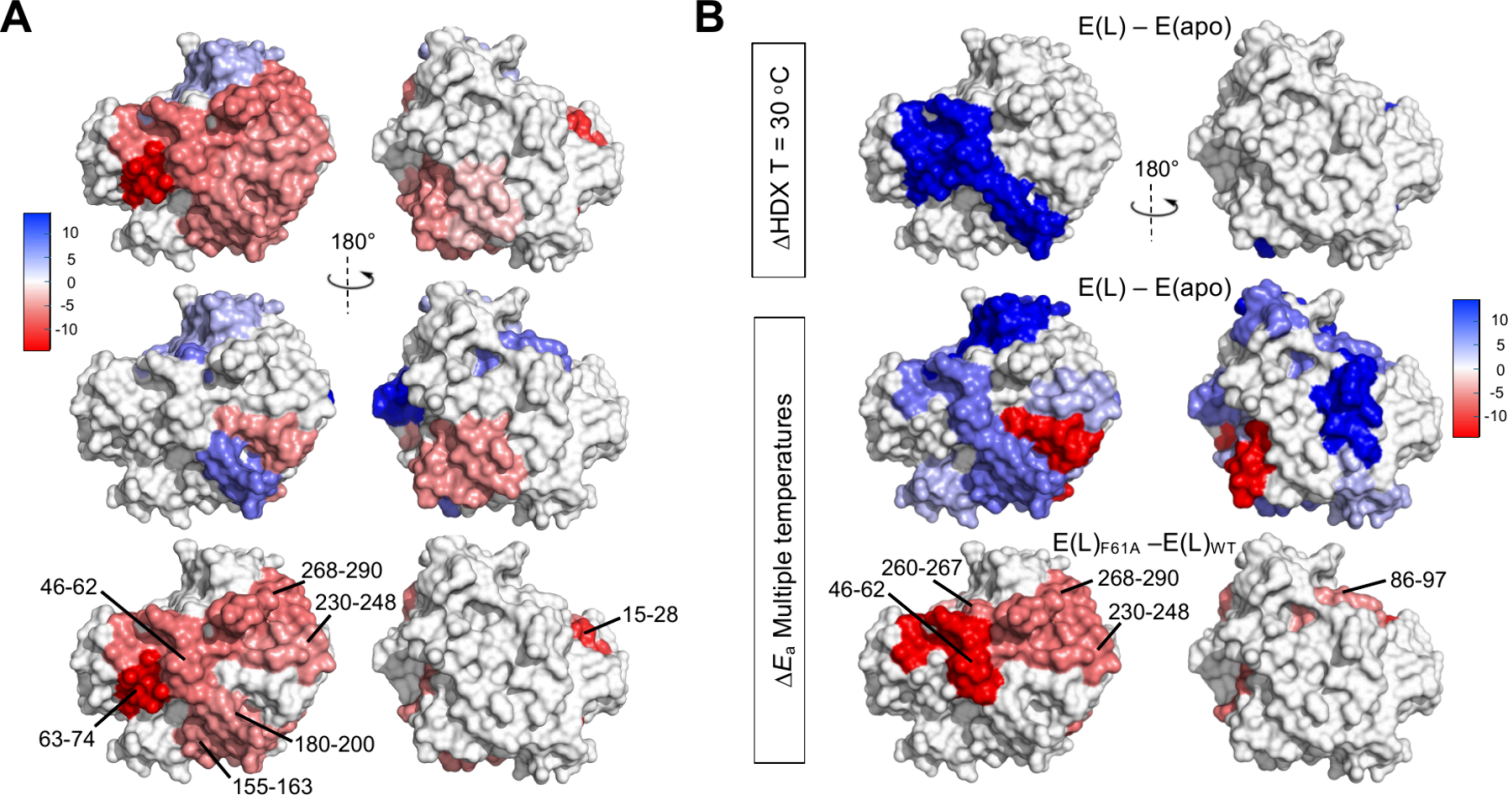
(A) Structural mapping of (top) Δ*E*_aHDX_(F61A-WT), (middle) Δ*E*_aHDX_(F61I-WT), and (bottom) the final refined thermal networks for apoenzyme, where heat maps of different colors define more flexible (red tones) or more rigid (blue tones) regions.^54^ (B) Structural mapping of the impact of the tight binding inhibitor pentostatin on HDX-MS of mADA: (top) single temperature HDX-MS, showing specific region (blue) protected by bound inhibitor, (middle) TDHDX showing regions that change protein flexibility in the E(L) complex relative to apoenzyme, and (bottom) the final thermal networks captured for mADA in complex with its TSA analogue.^125^ Panel (B) reproduced from ref (125), available under a CC-BY 4.0 license. Copyright 2022 Gao et al.

mADA is a paradigmatic TIM barrel enzyme that hydrolyzes adenosine to inosine and ammonia (Figure 4). In this case, our studies employed a series of variants (Ile, Leu, Val, Ala or Gly) at a single position, Phe61, located within a hydrophobic wall immediately behind the purine ring of a bound inhibitor (Figure 4A).^54^ As for SLO, the impact of reductions in side chain volume on *k*_cat_ (water addition to the purine ring is rate-limiting^122,123^) and *E*_a_(*k*_cat_) was investigated. The variants exhibit a similar reduction in *k*_cat_ (∼15-fold at 30 °C) accompanied by a linear increase in *E*_a_(*k*_cat_) from 11.5 to 19.6 kcal/mol for WT to F61G (Figure 4B). TDHDX studies were performed on the WT mADA, F61A (*E*_a_(*k*_cat_) = 17.3 kcal/mol) and a control variant, F61I for which *E*_a_(*k*_cat_) is unchanged from WT mADA. Comparing 3-D maps of regional differences in *E*_a_(*k*_HDX_) between (i) F61A and WT mADA (Figure 5A, top) and (ii) F61I and WT mADA (Figure 5A, middle) indicates impacts from F61I that are distinct from changes in protein flexibility that alter *E*_a_(*k*_cat_). These regions were therefore excluded from the final thermal networks in mADA (Figure 5A, bottom).^54^

The described TDHDX investigations used unliganded forms of SLO and mADA, raising the possibility that binding a substrate analogue would generate a different thermal network. This was addressed for m-ADA using pentostatin, a tight binding analogue for which enzyme–inhibitor X-ray structures are available.^124^ HDX-MS measurements in the presence of pentostatin at a single temperature show protection within the position of bound inhibitor that reaches across the interior bowl of the TIM barrel, with no detectable impact on the reverse face of the barrel (Figure 5B, top).^125^ TDHDX measurements for the mADA/pentostatin complex indicate an increase in the energy of activation (Δ*E*_a_) for local protein mobility throughout a large portion of the enzyme (Figure 5B, middle), consistent with a widespread decrease in global protein flexibility in the liganded form. We then compared TDHDX behavior in the WT mADA/pentostatin and F61A variant/pentostatin complexes. The resulting difference map (Figure 5B, bottom) retains the major features seen in apo-mADA (Figure 5A, bottom); two thermal networks reach from opposite protein/water surfaces toward the site of substrate binding. The change in flexibility within the pocket that binds the ribose ring of substrate is no longer detected when pentostatin is bound.^125^ Evidence for including this latter segment in the thermal network of the apoenzyme had been tentative, however, suggesting that it plays a relatively minor role in energy transfer from solvent to active site.^54^

### 4.3 Comparative Analyses of Four TIM Barrel Enzymes

**Figure 6:**
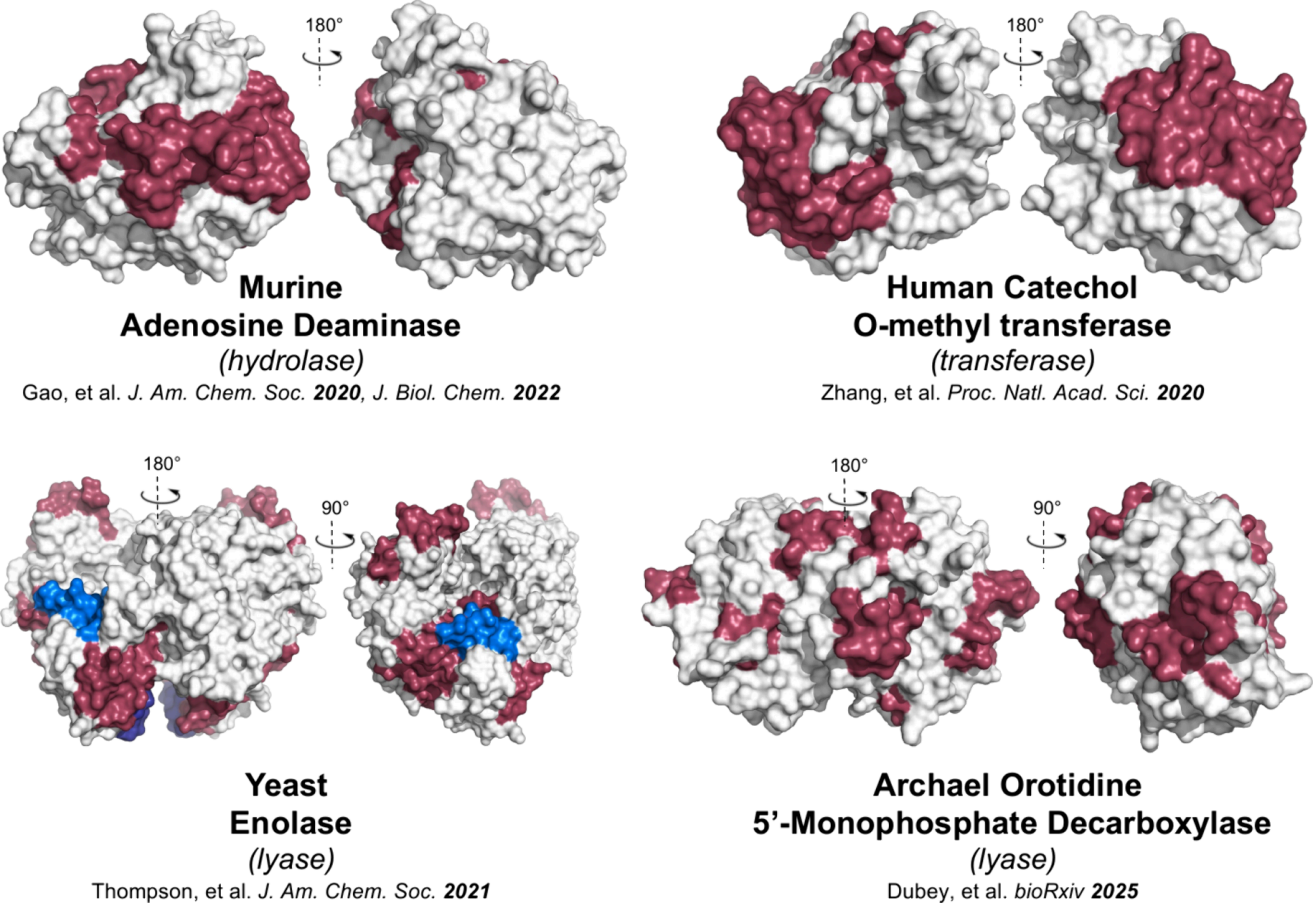
Thermal energy network comparison among TIM barrel enzymes. The final TDHDX-derived energy transfer pathways in mADA,^54,125^ yeast enolase,^126^ and human COMT^127^ are found to be comprised of two distinct networks. For archaeal OMPDC that catalyzes one of biology’s most challenging reactions, four synergistic thermal energy networks have been identified. In every instance, the revealed networks converge at the reactive bond of substrate within the respective active site.^106^

The functional diversity of TIM barrel enzymes offers the opportunity to explore whether one, or more, thermal networks within a conserved protein scaffold are conserved across different chemical reactions.^111^ We therefore extended our TDHDX investigations to three other members of the superfamily: yeast enolase,^126^ human catechol-O-methyltransferase (COMT),^127^ which is a primitive TIM-barrel enzyme, and a thermophilic orotidine 5′-monophosphate decarboxylase (OMPDC).^106^ These enzymes have different oligomeric structures; mADA and COMT act as monomers whereas enolase and OMPDC are functional dimers. Their metal dependencies also differ: mADA relies on a single tightly bound Zn ion, enolase and COMT utilize Mg ion, and OMPDC functions without metals. Among these four enzymes, enolase undergoes a large loop closure upon substrate and Mg^2+^ binding, and OMP binding organizes a loop in OMPDC.^128^ TDHDX-derived networks are evident in all four apo-enzymes under conditions where turnover is (at least) partially rate limited by a chemical step. Each thermal network is unique to the reaction catalyzed by its cognate enzyme, not to the generic TIM barrel fold (Figure 6).

All four networks are more complex than that determined for SLO (Figure 3D). We suggest this is a consequence of the relative ease of cleaving the activated C–H bond of linoleic acid compared with catalyzing the diverse chemistry of the four TIM barrel enzymes.^106^ Remarkably, the number of thermal networks present in each enzyme increases from one (SLO) to two (m-ADA, enolase and COMT) to four (OMPDC) (Table 2). For OMPDC, Δ*H*^⧧^(*k*_cat_) is reduced by 26 kcal/mol relative to that of the corresponding, uncatalyzed solution phase reaction (Table 1),^49,50^ an indicator of the extremely challenging decarboxylation reaction that OMPDC has evolved to catalyze.

**Table 2:** Summary of SLO and TIM Barrel Enzymes with TDHDX-Derived Pathways for Thermal Energy Transport from Solvent to Active Site

Enzyme	Structural features of thermal network(s) (TN)	Ref
m-ADA	Two networks reach from opposite faces of the protein toward the active site comprised of Zn^2+^, its ligands and catalytic acid and catalytic base side chains. At the left-hand face, a region of network reaches from a solvent exposed loop containing Lys54 to a helix turn helix motif that presents a hydrophobic wall to the backside of bound substrate (Figure 5).	(125)
SLO	A single “ILV-based network” connects surface loops (peptides 284–299 and 317–334) to Leu546, a “keystone residue” that contacts bound substrate (Figure 3).	(117)
Enolase	Two parallel networks link separate protein/solvent interfaces to the active site. One terminates at the substrate C–H that undergoes deprotonation, the other at an active site loop that creates a second Mg^2+^ binding site upon closure. This flexible loop itself is not part of an identified network.	(126)
COMT	Two networks are oriented at an angle of ca. 90°. The smaller network reaches from solvent toward Tyr68, which sits above the sulfur-bearing methyl group of SAM. The second, larger network extends from behind the methyl-bearing sulfur of SAM to the entire region surrounding the bound cofactor.	(127)
OMPDC	OMPDC displays four discernible pathways for thermal energy transfer by TDHDX measurements: TN-1 reaches from solvent to the phosphate of bound substrate; TN-2 interacts with the ribose ring of bound substrate; TN-3 connects TN-1 to TN-2 and may facilitate their synergistic activation; and TN-4 includes the catalytic loop 5 containing Ser127 (proposed to stabilize a vinyl carbanion/carbene intermediate) and the mutational site Leu123, which is part of a hydrophobic cluster (proposed to facilitate CO_2_ release).	(106)

## 5 Generating Models for the Transfer of Energy from Solvent to Active Site

- Temperature-dependent Stokes shift measurements at protein/water interfaces reveal enthalpic barriers for environmental reorganization identical to those of chemical transformations in the active site.
- The different magnitudes of *k*_Stokes shifts_ and *k*_cat_ arise from differing dependencies on a common, long-range and transient (<ns-ps) environmental restructuring.

### 5.1 Temporal Resolution of Motions within Identified Thermal Networks: Time- and Temperature-Dependent Stokes Shift Measurements

**Figure 7:**
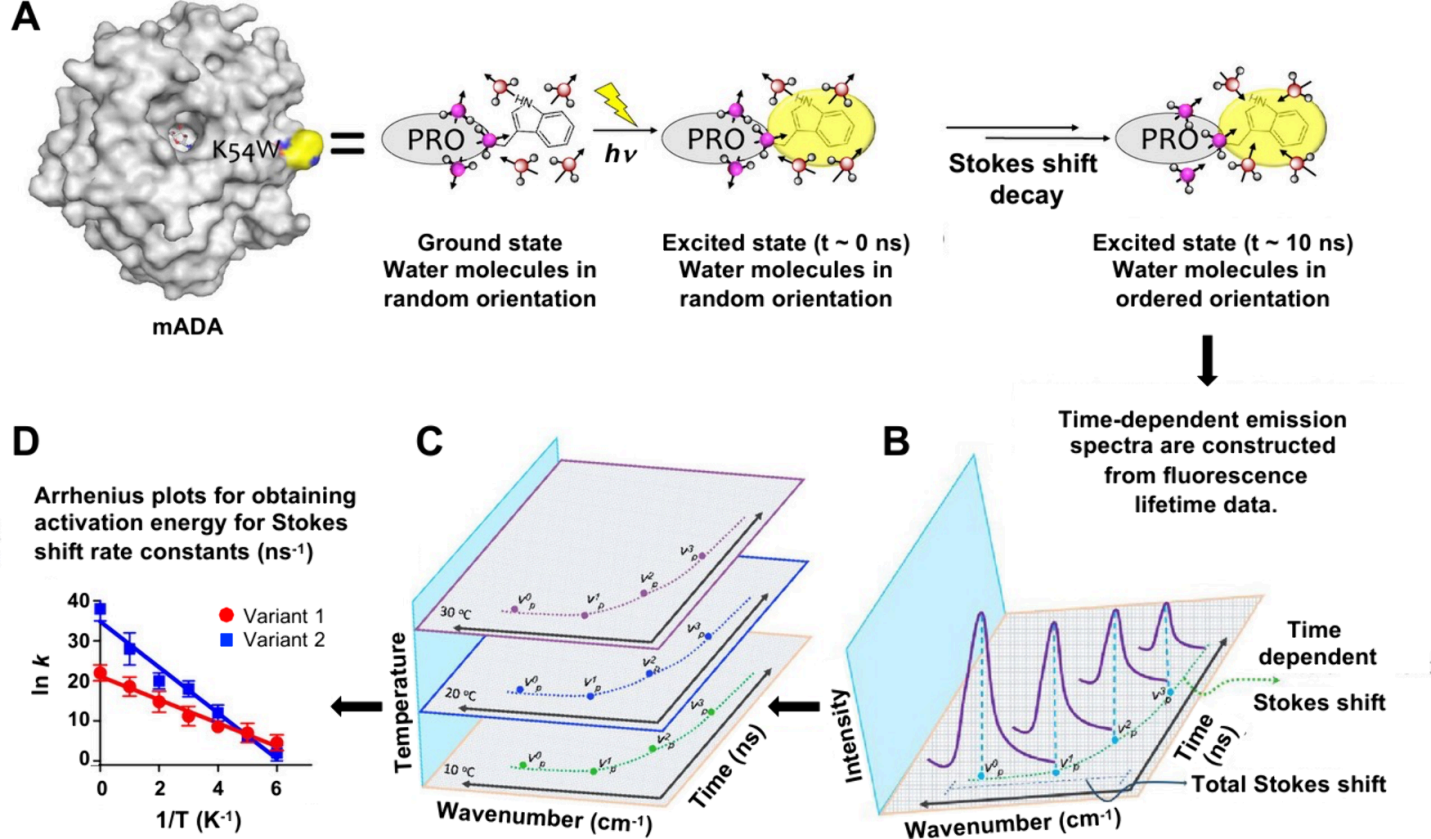
Stokes shift measurements: (A) Illustrated for mADA (represented as PRO), where a single tryptophan is inserted at the protein surface. Free and protein-bound waters are colored pink and purple. Configurations are similar in the ground state and immediately after photoexcitation (*t* ≈ 0 ns). With increasing time, free waters and protein surface residues move into altered configurations, to accommodate dipolar changes induced in the photoexcited state chromophore. (B) Using fluorescence lifetime data collected at multiple emission wavelengths, spectra are seen to shift to longer wavelengths (smaller wave numbers) with some decrease in the fluorescence intensity. (C) These data allow the construction of time-resolved emission spectra (TRES) as a function of temperature. (D) Fitting of data in (C) to the Arrhenius equation leads to the activation energy, *E*_a_(*k*_Stokes shift_). Reproduced with permission from ref (130). Copyright 2024 American Chemical Society.

The ability of TDHDX to spatially locate thermal networks linking solvent to the active site “sets the stage” but fails to reveal the process whereby protein scaffold-based reorganization converts ground state, ES complexes to their corresponding activated complexes. We therefore turned to temperature-dependent Stokes shift (TDSS) measurements,^117,129,130^ employing either intrinsic or extrinsic fluorescence probes to report on rapid protein structural changes (ns–ps) relevant to barrier crossing in the activated complex (fs). Once again, our focus was on measuring activation energies, i.e. correlating the temperature dependence of rate constants for active site chemistry and for solvent/scaffold reorganization. The principles of and protocols for these Stokes shift studies have been widely reviewed (Figure 7).^131−136^

Briefly, a probe placed at a specific protein/solvent interface undergoes photoexcitation, leading to an excited state with altered dipolar properties. The photoinduced change in charge distribution causes the protein and solvent surroundings to undergo a time-dependent restructuring that stabilizes the excited state. In the process, λ_max_ for light emission from the probe moves to longer wavelengths in a time-dependent fashion with a rate constant *k*_Stokes shift_. Repeating the excitation over a range of temperatures provides *E*_a_(*k*_Stokes shift_).

#### 5.1.1 Example 1: SLO

**Figure 8:**
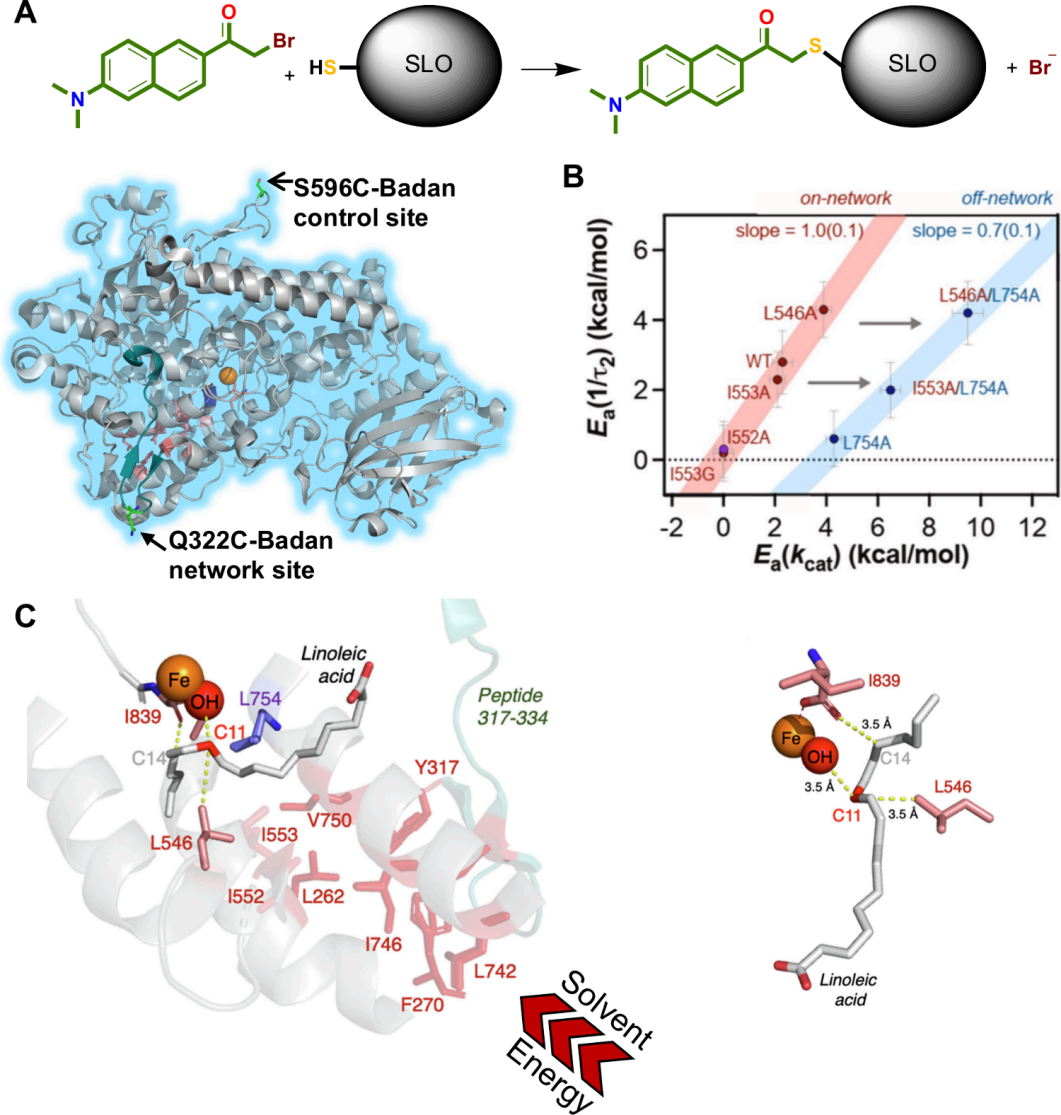
Stokes shift measurements of Badan-labeled SLO. (A, top) Labeling of SLO via reaction of an engineered surface cysteine with an activated bromo-derivative of Badan. (A, bottom) Ribbon diagram of SLO showing two separate engineered cysteine sites.^129^ (B) Activation energies for measured Stokes shifts (*y*-axis) vs activation energies for catalysis (*x*-axis) indicate a 1:1 relationship for on-network residues (red line). The insertion of L754A (blue line) changes *E*_a_(*k*_cat_) but not *E*_a_(1/τ_2_).^117^ (C, left) Active site modeling shows the proximity of the reactive carbon (C11) of substrate to the Fe-OH. The C14 of LA (as shown), or C8 (in an alternate binding mode with carboxylate pointing out), binds proximal to the carboxylate of Fe ligand I839. Panels (B) and (C) reproduced with permission from ref (117). Copyright 2023 PNAS.

Our initial Stokes shift measurements were performed on SLO. Although tryptophan side chains are suitable probes for such experiments, the large number of these residues in native SLO precluded their use. It was, however, possible to attach the extrinsic fluorescent probe BADAN^135^ at single site cysteine residues introduced to solvent-exposed regions of SLO. Two sites were chosen for probe attachment: Cys322 and Cys596 via the Q322C and S596C variants.^129^ Cys322 lies at the solvent-exposed end of the thermal network (Figure 8A) while Cys596 is located in a distal surface region of the enzyme that is not involved in functionally related energy transfer. In both instances, biexponential decay curves were observed for *k*_Stokes shift_, with average lifetimes of ca. 3 ns. Significantly, the temperature-dependent behavior of *k*_Stokes shift_ gave an *E*_a_(*k*_Stokes shift_) close to zero when BADAN was attached to Cys596, whereas *E*_a_(*k*_Stokes shift_) for the probe at Cys322 was *almost identical* to *E*_a_(*k*_cat_). This was an unexpected result, because catalysis and Stokes shifts represent completely different physical processes taking place on highly divergent time scales.^129^

Cysteine was then introduced in place of Gln322 for the series of SLO variants with altered *k*_cat_ and *E*_a_(*k*_cat_) values, and each double mutant was labeled with BADAN. Temperature-dependent Stokes shift analyses on this series of variants corroborated the 1:1 identity between *E*_a_(*k*_Stokes shift_) and *E*_a_(*k*_cat_) for “in network” residues (Figures 3C and 8B). By contrast, mutating Leu754, a residue that lies outside of the thermal network, increased *E*_a_(*k*_cat_) but left *E*_a_(*k*_Stokes shift_) unaltered (Figure 8B). These data (the first of their kind), *indicate that two disparate processes, at sites separated by a distance of ca. 20 Å, are initiated by a site-specific, shared thermal activation event.* The rate of activation must exceed the faster of the two measurements (ns) and is therefore ascribed to collective protein motions in the subns to ps regime.

A computational model^82^ of linoleic acid (LA) bound in the X-ray structure of apo-SLO was used to understand how a thermal network, beginning at the enzyme surface and terminating at Leu546, would generate the tunneling ready states in C–H activation. The reactive carbon (C11) and more remote C14 of LA lie within van der Waals distances of Leu546 and the Ile839 carboxylate (6th ligand of the active site Fe), respectively (Figure 8C).^137,138^ ENDOR measurements on Mn-substituted SLO independently established that C11 of the substrate is 3.1 Å from a bound water/hydroxide at the metal cofactor.^114^ Two properties must be optimized if the ground state structure is to achieve active site hydrogenic wave function overlap. First, the distance between hydrogen donor (C11 of LA) and acceptor (oxygen of Fe(III)-OH) must approach ca. 2.7 Å.^34^ Second, the reactant/product potential energy wells must become transiently degenerate. We suggest that both changes are achieved simultaneously by energy transfer through the thermal network, which facilitates translation of four substrate carbons (C11–C14) closer to the Fe(III)-OH moiety. Reducing the distance between the hydrophobic methylene of C14 and the Ile839 carboxylate alters the local electrostatics, possibly via changes in H-bonding between Ile839 and the hydroxyl group in Fe(III)-OH.^139^ Future computational studies will be needed to examine whether these factors alone change Δ*G*° for the interconversion of substrate and the pentadienyl radical (estimated to be −5 kcal/mol) to a value compatible with transient wave function overlap.

At a minimum, any physical process capable of initiating this transient reconfiguring of the substrate requires a stable configuration behind the bound substrate (possibly provided by Leu754 and surrounding residues outside of the thermal network) and a mobile element near the protein water interface. In SLO, this element corresponds to the intertwined loops 1 and 2 located at the end of the thermal network (Figure 3B); Cys322, located midway between these two loops, is the BADAN-labeled side chain characterized by TDSS (Figure 8A).

#### 5.1.2 Example 2: mADA

**Figure 9:**
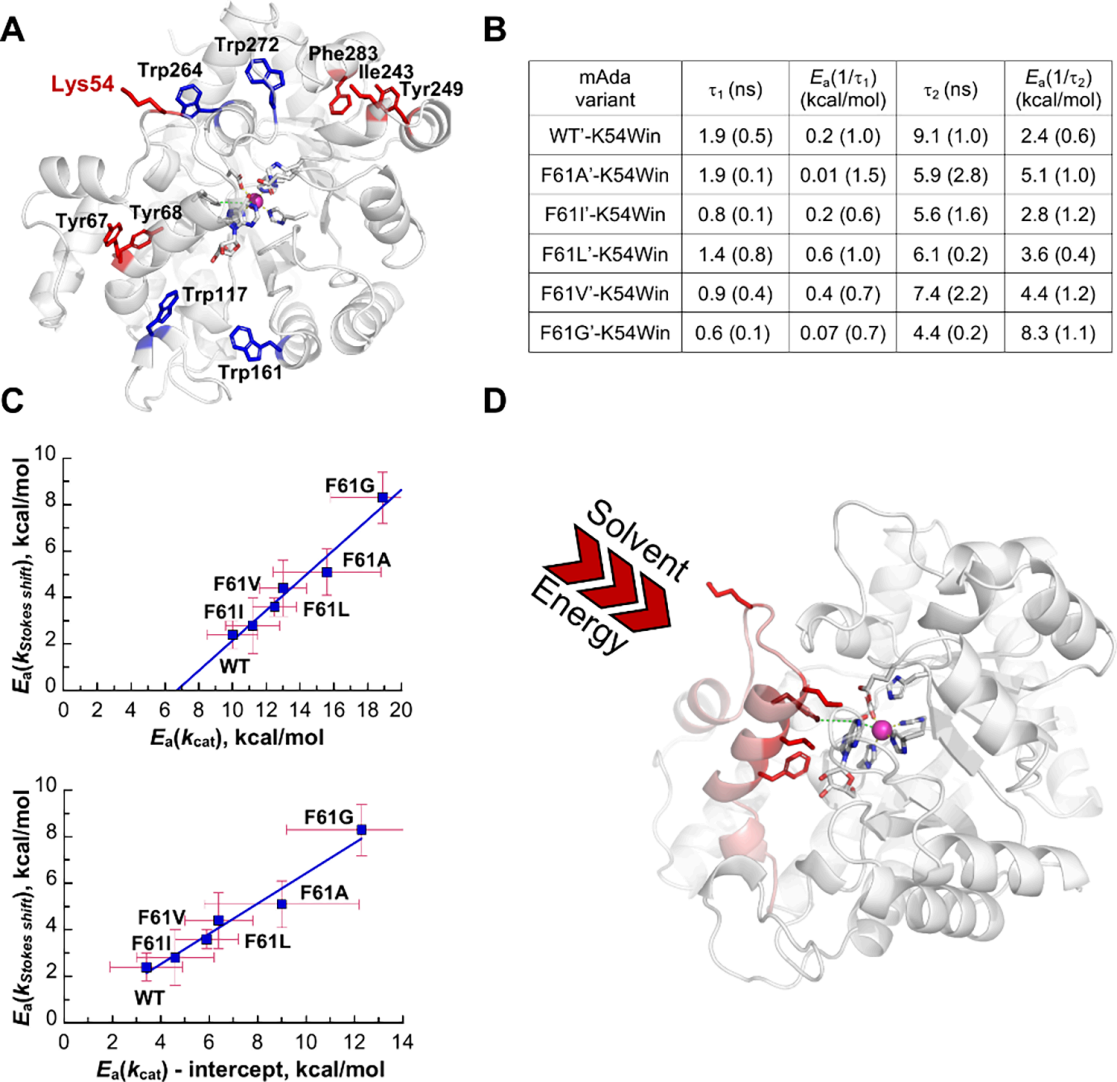
Stokes shift measurements of single tryptophans engineered into a Trp-free form of m-ADA (WT′). (A) The label blue indicates positions where single Trps were back inserted as controls, and red indicates positions tested for possible correlations with *E*_a_(*k*_cat_). K54W was chosen for in depth study. (B) Lifetimes (*T* = 30°) and activation energies for Stokes shifts within the F61X’ K54W series.^130^ (C) Plots of *E*_a_(*k*_Stokes shift_), representing 1/τ_2_ from the above Table, vs *E*_a_(*k*_cat_). The top graph shows raw data with a nonzero intercept; correction for this leads to a close to 1:1 relationship with *E*_a_(*k*_cat_).^130^ (D) Regions of WT mADA structure labeled red to represent a wall of hydrophobic residues in contact with bound substrate and a connected surface loop containing the exposed K54. Solvent activation at the K54 loop is ascribed to ca. half of the total energy transfer from solvent. Adapted from ref (130). Copyright 2024 American Chemical Society.

We next sought to determine whether behavior similar to SLO could be detected for enzyme reactions that require the coordination of multiple active site components. Within the defined substrate binding region of mADA are three key catalytic residues: a general acid (Glu217), a general base (His238) and a Zn(II) ion, which is coordinated by water, three histidines and an aspartate.^110,140^ As discussed above, the thermal network in mADA is comprised of two regions that reach from opposing solvent/protein interfaces toward the active site (Figure 5B, bottom). Efforts to introduce BADAN following the strategy developed for SLO failed due to protein denaturation. As an alternative, we turned to studies of intrinsic tryptophan fluorescence,^141^ and began by preparing a tryptophan-free variant of mADA (WT′).^130^ The WT′ mADA variant was stable and its catalytic properties were preserved relative to native protein, making it an excellent scaffold for constructing a range of single tryptophan variants. In the WT′ mADA/K54W double variant, the tryptophan probe resides on a loop proximal to the hydrophobic wall in communication with bound substrate. Tryptophan was also inserted at four control positions (residues 117, 161, 264 and 272) located outside of the identified thermal network (Figure 9A).^130^

In order to examine the relationship between *E*_a_(*k*_cat_) and *E*_a_(*k*_Stokes shift_), a series of double mutants were generated from the WT′ mADA/K54W variant using the same series of Phe61X replacements employed in the TDHDX experiments (Figure 4B). The results for these K54W/F61X double variants (Figure 9B) were fit by a biexponential process with ns relaxation times of τ_1_ and τ_2_ that differ in magnitude by 5 to 10-fold and in their sensitivity to temperature. Distinct from control positions, the magnitude of τ_2_ is found to be temperature-dependent and yields values of *E*_a_(*k*_Stokes shift_) that vary with the size of the side chain at position 61 (Figure 9C). These data show two notable features: there is a linear correlation between the measured values for *E*_a_(*k*_cat_) and *E*_a_(*k*_Stokes shift_) in the double variants and the line intersects the *x*-axis at a nonzero value of ca. 6.5 kcal/mol (Figure 9C). Recalling that mADA contains two separate thermal networks, the Trp54 probe reports on only one of the two potential pathways for energy transfer from solvent to active site. The presented data argue that only 43% [(11.5–6.5)/11.5] of the total activation energy needed to generate the tetrahedral intermediate by reaction of zinc-bound water with substrate in the WT′ mADA variant is transferred to the active site via the left-handed network. Analyzing the data to reflect this portion of the activation barrier, the *E*_a_(*k*_cat_)-intercept as a function of *E*_a_(*k*_Stokes shift_) gives a close to 1:1 relationship between the enthalpic barriers for two very different processes,^130^ analogous to the trends shown for SLO (Figure 8B).^117^

These findings corroborate our expectation that classical enzyme reactions follow the same patterns and rules as those deduced from studies of the tunneling enzyme SLO. Positioning a fluorescent probe in a solvent-exposed position within a functionally linked thermal network of mADA results in an almost identical activation enthalpy, accompanied by a much faster time scale (ns) for the observed relaxation than that for *k*_cat_ (ms). We propose that collisions with water molecules at the protein surface result in rapid and long-range protein restructuring through changes in vibrational excitations that transmit energy from solvent to the enzyme active site. Analogous to SLO, visual inspection of mADA implicates the pattern of *a mobile solvent-exposed loop that is in close proximity to the region(s) of the scaffold mediating site-specific energy transfer from solvent to active site*.

### 5.2 Why Rate Constants with Identical Energies of Activation Differ 10^6^-fold in Magnitude

An initially puzzling aspect of the Stokes shift studies for both SLO and mADA was the large difference (ca. 10^6^-fold) in the magnitude of *k*_Stokes shift_ and *k*_cat_ in spite of the strong similarity (or identity) of *E*_a_(*k*_Stokes shift_) and *E*_a_(*k*_cat_). A related discrepancy between the computed and measured rate constants for vibrationally non-adiabatic expressions for H-tunneling was also noted by Hammes-Schiffer and co-workers (Section 3.2).^99^ A generic form of eq 7 is thus given in eq 14, where *k*_tun_ is replaced by *k*, and corresponds to the shared thermally activated, site-specific protein environmental reorganization characterizing catalysis and Stokes shifts:
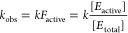
14

According to this formalism, the different magnitudes of the rate constants *k*_cat_ and *k*_Stokes shift_ reflect very large differences in the fraction of active enzyme that supports their distinctive physical processes. Stabilization of a photoexcited chromophore at a protein/water interface is capable of proceeding within a large fraction of the total enzyme population. In contrast, complex active site interactions involve a large number of active site components that are a prerequisite for successful conversion of reactant to product. In this case, *F*_active_ is expected to be significantly altered, resulting in a reduced *k*_obs_.

The impact of *F*_active_ on an observed rate constant is widely observed in enzyme kinetics.^142^ For example, many enzymes exhibit pH-dependent values of *k*_cat_.^143^ When protonation of an active site residue is essential for a reaction to occur, and the protonated (E-BH) and unprotonated (E-B) forms of the enzyme are in equilibrium, *F*_active_ = [1/(1 + *K*_H_/H^+^)] and *k*_obs_ = *k*/(1 + *K*_H_/H^+^). Here, *K*_H_ is the equilibrium constant that describes the acidity of the residue. In a related context, Åqvist and co-workers have shown that curvature in Arrhenius plots for temperature-dependent enzyme activity can arise from rapidly equilibrating on- and off-pathway forms of the enzyme.^47^

**Figure 10:**
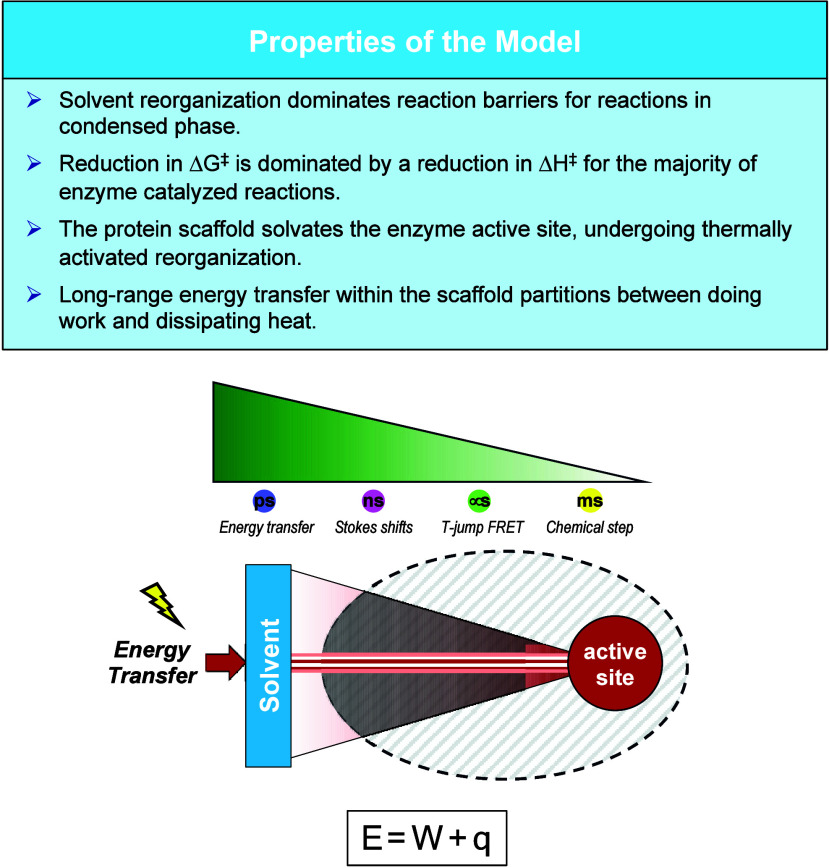
Site-specific energy transfer from a protein/solvent interface initiates remote enzyme chemistry. The key properties of a dynamical model are summarized (top). The image below indicates possible time scales for measurable responses to a rapid (ps), solvent-induced protein restructuring. The energy (E) derived from the kinetic and potential energy of solvent, undergoes a partitioning between productive work (W) and dissipation of heat (q). The response (green wedge) varies from ns for Stokes shifts (discussed herein for SLO and mADA) to μs for FRET behavior observed in an alcohol dehydrogenase.^144^ Enzymatic turnover occurs at the active site on a time scale of ms. Different physical events with widely differing rate constants can therefore share a common activation energy with variations in partitioning of productive work and heat loss.

Our working model for the process by which thermal networks facilitate barrier crossing involves a transfer of energy from solvent into a range of internal protein vibrational modes (Figure 10).^145^ For vibrational excitation of residues in an enzyme active site, collective, low frequency, and anharmonic vibrational coupling (in the THz range) may be necessary to ensure efficient energy transmission (∼20 Å^2^/ps) that minimizes heat loss.^146^ We note that relatively long distances between any solvent/protein interface, where initial energy transfer occurs, and the active site provide multiple channels for heat dissipation. In contrast, Stokes shifts for chromophores located near the site of initial energy transfer from solvent to protein will be less sensitive to ongoing heat loss. In consequence, although barrier crossing in processes represented by *k*_cat_ and *k*_Stokes shift_ both take place on a fs time scale, the differential success of each thermal activation event in achieving reaction-appropriate configurations (reflected in *F*_active_) introduces a second source of the large discrepancies in the experimental values of *k*_obs_.

## 6 A New Model for Understanding Enzyme Function

- Femtosecond barrier crossings are associated with a combination of preorganized enzyme–substrate ground states and anisotropic energy flow from solvent to active site.
- Site-specific energy conduits distinguish enzyme reactions from small molecule chemistry and from allosteric regulation.

### 6.1 Distinguishing Broadly Distributed Conformational Landscapes (Preorganization) from Thermally Activated Protein Networks (Reorganization)

Enzymes explore multiple, equilibrated, conformations in solution on a hierarchy of time scales,^42^ which must be accompanied by correlated changes in the position and orientation of water molecules in the first solvation shell.^147^ Substrate binding has been shown to perturb the distribution of substates present in the apoprotein by a process of conformational selection.^33,148,149^ Once formed, the ES complex continues to “wiggle and jiggle”, resulting in rapidly interconverting ground state structures, whose populations are defined by the Boltzmann distribution, and which are characterized by a range of rate constants for barrier crossing. This process of ground state ES equilibration is expected to be biased toward structures that are well organized for progression to product.^150,151^

This model, however, provides no molecular picture for energy flow within each Michaelis complex that converts productive ground states to activated complexes. The seminal work of Hynes,^35^ Kreevoy,^152−154^ and others,^31,155,156^ has shown the essential role of environmental reorganization within the solvent to create activated complexes capable of successful barrier crossings. According to the ideas developed in this Perspective, environmental reorganization at the active site is facilitated by collisions at specific regions of the protein/solvent interface, presumably through energy transfer to mobile surface loops. In conjunction with preorganized ES substates,^157,158^ reorganization results in highly efficient, conversions of substrate to product.^117^ This proposal is clearly at odds with the widely held belief that enzyme catalysis simply results from tight binding of transition state-like structures.

In principle, barrier crossings might be argued to arise from very rare, high energy substates within a distributed conformational landscape. Such a model, however, also needs to take into account the multiple TDHDX-based examples of spatially defined, thermally activated connections linking protein/solvent interfaces and key components in the active site (Figure 6). Further, the direct dynamical measurements that reveal *identical activation energies* for fluorescence relaxation of surface-appended probes and active site chemistry (Figures 8 and 9) provide strong evidence in support of the ideas presented herein (Figure 10).^117,129,130^

The phenomenon of anisotropic heat flow in proteins has been well established,^100,101^ rendering our evidence for *site-specific energy flow* within enzyme scaffolds less counterintuitive. Similar ideas^156^ were introduced into the literature in 1982 and then ignored, presumably due to the lack of supporting data. We view thermal networks as a lens that concentrates energy where it is needed to enable productive barrier crossings. The utilization of anisotropic energy flow side steps the far less efficient, isotropic energy transfer that occurs in small molecule reactions (Figure 10). Ongoing theoretical work is suggesting possible mechanisms for a focus of solvent-derived energy in protein regions proximal to reacting bonds.^159−161^

### 6.2 Distinguishing Thermal Activation of Active Site Chemistry and the Mechanism of Allosteric Regulation

Here we briefly compare the characteristics of thermal networks for facilitating enzyme chemistry with allosteric regulation of proteins. Qualitatively, the two processes appear similar in that they require communication between remote sites via specific protein networks, but in fact the two processes are quite distinct albeit potentially complementary. Perhaps the most critical difference is that transient environmental reorganization to allow barrier crossing is different from the shift in an ensemble of ground states that dominates allostery.^162,163^ As described above, active site chemistry (*k*_cat_) displays a reorganization energy identical to the reorganization energy taking place at an enzyme/solvent interface (*k*_Stokes shift_). In contrast, allosteric regulation occurs widely in proteins having catalytic or noncatalytic functions, the hallmark of allostery being an energetic coupling between the binding of two ligands at sites remote from one another.^163^ In many instances, these coupled changes in binding affinity are accompanied by clearly observable structural changes; however changes in protein dynamics, i.e., conformational sampling, may also occur upon effector binding without any discernible change in the averaged conformation from static structural methods.^164^ These properties indicate that allosteric effectors (activator or inhibitor) regulate function by altering populations within the equilibrated, ground state conformational ensemble of the protein.

Further, in the case of enzymes, binding of an allosteric effector most often alters *K*_m_ for the substrate by changing association or dissociation rate constants (*k*_assoc_ or *k*_dissoc_) while having no effect on *k*_cat_ (K-type allostery).^163,165^ Likewise, when *k*_cat_ is allosterically regulated (V-type allostery),^163,165^ the effect mainly arises from an altered rate of product dissociation rather than from changes in the rate constants for the chemical steps.^163,165^ Occasionally, the rate constant for a chemical step is increased by an allosteric activator due to an increased fraction of conformationally productive preorganized substates. For example, the overall transformation catalyzed by imidazole-glycerol-phosphate synthase is composed of two half reactions, at different active sites located in separate protein domains. Binding the second substrate in the remote site allosterically activates glutaminase activity (first half reaction) by increasing the fraction of enzyme in the ground state ensemble having a correctly preorganized oxyanion hole that is essential for catalysis.^166,167^ A related phenomenon has been documented for enzymes regulated by reversible phosphorylation.^168^ Phosphorylation of ERK2 kinase leads to a dramatic increase in rate constant for the chemical step,^169^ accompanied by a significant increase in active site structuring as deduced from MD modeling.^170^

## 7 Concluding Remarks

- Historically, enzyme structure was related to reaction mechanism using static protein structures.
- Barrier crossings depend on protein motions responsible for a combination of ground state conformational ensembles and environmental reorganization.
- A role for anisotropic thermal energy transport introduces a new dimension into the quest for improved catalyst design.

**Figure 11:**
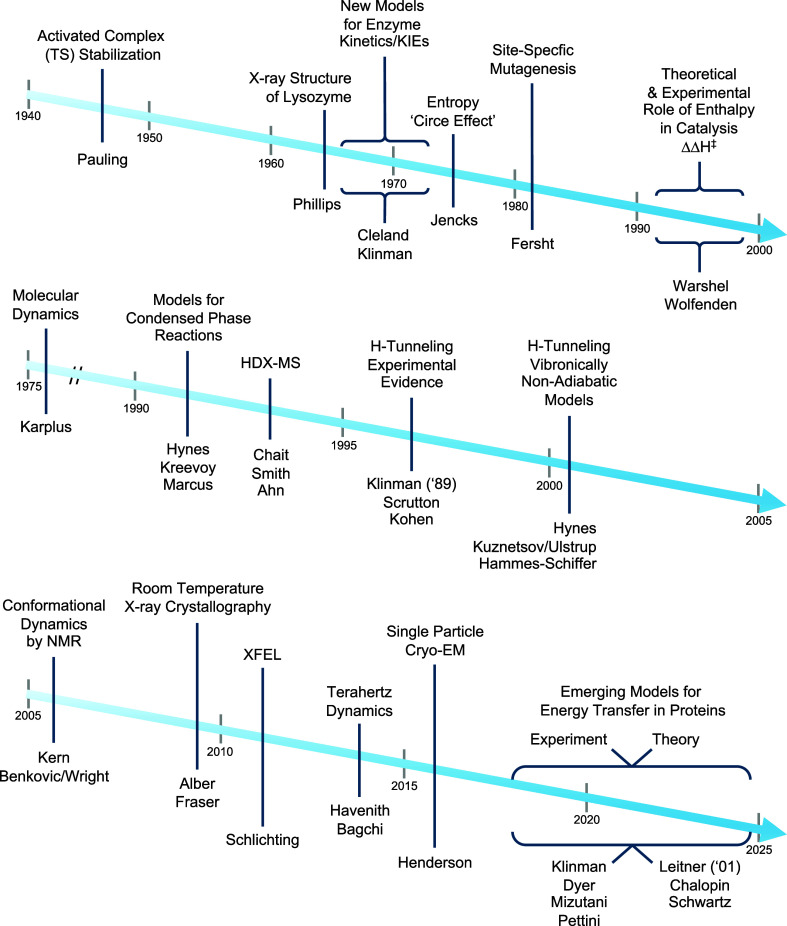
An 80 year trajectory of enzymology. Arrow 1: The early timeline represented a highly focused deduction of enzyme mechanism from static structures, kinetic methods and theory. Arrow 2: MD, followed by new models for condensed phase reactivity and HDX-MS, set the stage for the modern era; H-tunneling established a role for DAD sampling in barrier crossings. Arrow 3: New biophysical tools, together with theoretical advances, indicate a synergistic combination of conformational ensembles and energy transfer pathways from solvent to active site in the transitioning of ES ground states to activated complexes.

Our understanding of enzyme function has always been driven by new technologies and experimental findings (Figure 11). Following from Pauling’s hypothesis,^1,2^ insights into chemical bonding and functional group reactivity from physical organic chemistry were eagerly applied to enzyme mechanisms,^171−173^ and the subsequent development of protein X-ray crystallography was a game changer^174^ allowing visualization of active sites and the details of intra- and intermolecular interactions.^175^ New experimental methods, coupled with a firm mathematical understanding of reaction kinetics,^176^ gave insights into the order of substrate binding/product release in multicomponent reactions, and single-turnover methods provided rate constants for chemical steps.^177^

Similarly, the introduction of KIE measurements allowed identification of conditions where chemical steps could be isolated and studied,^178,179^ and quantification of bond order changes at the transition state.^180−182^ Jencks recognized the large reduction in the entropic contribution to reaction barriers that arises from preorganization of multiple functional groups within the active site upon protein folding.^37^ Finally, site-specific mutagenesis^183^ and advanced computational methods, such as EVB^184^ and QM/MM,^185^ have made it possible to test mechanistic proposals both qualitatively and quantitatively.^186^ These approaches confirm the importance of electrostatics for enzyme catalysis,^66^ as implied by the finding that enzymes primarily lower enthalpic barriers to reaction (Table 1).^19^

These breakthroughs occurred largely by viewing the protein scaffold as a static entity. MD simulations clearly show, however, that “enzymes are moving all the time over a wide range of time scales”.^42^ The demonstration of tunneling in enzyme-catalyzed hydride transfer also pointed the way to models in which enzymes use explicit motions to effect their function.^84,187^ With the extension of Marcus theory to enzyme-catalyzed reactions,^28^ alternate perspectives of active site reactivity developed,^79,153^ including models emphasizing a role for protein motions that directly alter chemical outcomes.^87,188,189^ At the same time, technological advances in X-ray crystallography and NMR enabled direct observation of rapidly equilibrating conformational landscapes.^190,191^ On the other hand, there remains resistance to dynamical models, and it has been argued that these make no significant contribution to enzyme catalysis.^16,192^

Our discovery of anisotropic thermal networks, using HDX-MS^103,105^ as a function of temperature TDHDX-MS,^107^ connects protein/solvent interfaces to active sites and provides a new perspective for understanding and manipulating enzyme function. The ability of such networks to enable rapid energy flow over long distances, as inferred from Stokes shifts measurements,^117,130^ raises many questions concerning the molecular mechanism(s) underlying energy transfer. Importantly, new methods to test this model are available including RT X-ray methods,^191^ free electron lasers to visualize protein motions on very rapid time scales,^193^ and the detection of transient intermediates by cryo-EM.^194−196^ Moreover, experimental and theoretical investigations of energy flow and dissipation through peptides and proteins are under active development,^197−199^ with methods to monitor low-frequency vibrational modes within protein scaffolds being of particular interest because these are anticipated to dominate rapid, cooperative and long-range changes in protein structure.^131,136,200^ Transition path sampling, which captures fs motions at the enzymatic transition state,^201^ is another demonstrated approach for identifying networks of interacting residues relevant to function.^202^ Understanding the molecular underpinnings of how protein scaffolds can initiate energetic excursions from enzymatic ground states to activated complexes has the capability to transform the face of enzymology, providing physical parameters and guidelines for *de novo* catalyst design.^203,204^ With the accumulation of a suitable database of the range of possible structural compositions for thermal networks, their prediction may become possible using computation and AI methodology.^205^
